# New Perspectives on Music in Rehabilitation of Executive and Attention Functions

**DOI:** 10.3389/fnins.2019.01245

**Published:** 2019-11-19

**Authors:** Yuko Koshimori, Michael H. Thaut

**Affiliations:** Music and Health Research Collaboratory, Faculty of Music, University of Toronto, Toronto, ON, Canada

**Keywords:** executive function, attention processes, music neuroscience, music improvisation, Neurologic Music Therapy

## Abstract

Modern music therapy, starting around the middle of the twentieth century was primarily conceived to promote emotional well-being and to facilitate social group association and integration. Therefore, it was rooted mostly in social science concepts. More recently, music as therapy began to move decidedly toward perspectives of neuroscience. This has been facilitated by the advent of neuroimaging techniques that help uncover the therapeutic mechanisms for non-musical goals in the brain processes underlying music perception, cognition, and production. In this paper, we focus on executive function (EF) and attentional processes (AP) that are central for cognitive rehabilitation efforts. To this end, we summarize existing behavioral as well as neuroimaging and neurophysiological studies in musicians, non-musicians, and clinical populations. Musical improvisation and instrumental playing may have some potential for EF/AP stimulation and neurorehabilitation. However, more neuroimaging studies are needed to investigate the neural mechanisms for the active musical performance. Furthermore, more randomized clinical trials combined with neuroimaging techniques are warranted to demonstrate the specific efficacy and neuroplasticity induced by music-based interventions.

## Introduction

Brain and biomedical research involving music has shown that music is a highly structured auditory language engaging complex perception, cognition, and motor control in the brain (Peretz and Zatorre, 2005; Stewart et al., 2006; Alluri et al., 2011; Lee et al., 2011; Thaut et al., 2014) and has a distinct influence on the brain by stimulating complex cognitive (Leggieri et al., 2019), affective (Koelsch, 2014), and sensorimotor processes (Thaut et al., 2015; Crasta et al., 2018; Schaffert et al., 2019).

As musical sound patterns are dynamic, continuously unfold, and change in time, external auditory input may serve well for cognitive rehabilitation, in particular for the domains of executive function (EF) and attentional processes (AP) by helping to adapt to a changing environment. Music by its nature drives attention exogenously (Klein and Lawrence, 2011; Thaut and Gardiner, 2014). Furthermore, music is a multidimensional stimulus consisting of multiple acoustical elements (e.g., pitch, loudness, tempo, rhythms, timbre, melody, harmony) that exist in temporally patterned simultaneity and sequentiality to drive AP (Thaut and Gardiner, 2014). In addition, active music performance involves moment-to-moment feedback systems, which facilitates monitoring, adjusting, and updating (Gardiner and Thaut, 2014). Further, there is some evidence that musicians perform better on EF/AP (e.g., Hanna-Pladdy and Mackay, 2011) as well as there are prefrontal structural and functional differences between musicians and non-musicians (e.g., Fauvel et al., 2014; Groussard et al., 2014). Furthermore, research shows that active musical performance extensively modulates the prefrontal activity in musicians (Erkkinen and Berkowitz, 2018).

Therefore, this sensory language may effectively be used as a therapy to induce neuroplasticity in the brain affected by disorders, diseases, and injuries (Särkämö et al., 2014). With this neuroscientific knowledge, music may be able to yield more specific therapeutic and stimulation outcomes for targeted functions. In this paper, we first briefly summarize existing studies investigating the effects of formal musical training on the EF/AP performance as well as on brain structure and function. Second, we discuss the neuroimaging/neurophysiological studies demonstrating the prefrontal neural correlates and neuroplasticity of active musical performance. Third, we present the body of literature in music-based interventions in healthy and clinical populations, focusing on the EF/AP findings. Finally, we present the future directions of research in this field to move forward the neuroscientific approach, which facilitates further advancement of music-based interventions for the EF/AP stimulation and rehabilitation.

## Music Training on EF/AP and Brain Structure and Function in Musicians

Some correlational and cross sectional studies have supported the beneficial effects of music training on EF/AP in older musicians (Hanna-Pladdy and Mackay, 2011; Hanna-pladdy and Gajewski, 2012; Liu et al., 2012; Amer et al., 2013; Moussard et al., 2016; Strong and Mast, 2019), younger musicians (Moradzadeh and Blumenthal, 2015; Okada and Slevc, 2018; Medina and Barraza, 2019), and musically trained children (Zuk et al., 2014). In addition, several studies demonstrated structural and functional changes associated with formal musical training in the prefrontal regions. For example, greater gray matter volume was associated with increasing musical practice in the multiple regions including supplementary motor area (SMA), superior/middle and medial frontal cortex, and insula (Groussard et al., 2014; James et al., 2014). Furthermore, resting-state functional connectivity revealed significantly greater connectivity density in the medial and lateral prefrontal regions as well as temporoparietal junction in musicians (Fauvel et al., 2014; Luo et al., 2014; Klein et al., 2016; Tanaka and Kirino, 2016).

A few studies investigated the brain activity during an EF task in musicians. One EEG study using a visual go/no-go task demonstrated that a group of 17 older musicians exhibited a significantly larger difference in the N2 amplitude (reflecting a conflict detect signal or inhibition of a prepotent response) in the central midline sites between go and no-go conditions compared to a group of 17 older non-musicians (Moussard et al., 2016). The music group also performed significantly better on the task, demonstrated with fewer no-go errors. Further, the music group showed a significant correlation between the N2 amplitude and the no-go task performance. However, there were no significant correlations between the N2 amplitude and any measures of musical background (age of first instruction, years of musical instruction, and hours of current practice). On the other hand, the measures of musical background, but not the task performance were significantly associated with the P3 amplitude that showed no significant group difference in amplitude but differential topography between groups (anterior shift in musicians). Two other studies demonstrated differential brain activities during EF tasks in younger musicians (Moreno et al., 2014) and musically trained children (Zuk et al., 2014) compared to non-musician groups without showing task performance differences between groups.

In summary, the cross-sectional or correlational studies in musicians have shed light on the potential benefit of formal musical training on EF/AP and brain changes in the prefrontal area. The task-based studies consistently demonstrated that individuals with formal musical training have differential brain activity during EF tasks in relative to those without it. However, these studies do not allow to determine the direct and causal effects of music training on EF/AP and brain changes. Furthermore, in these musician studies, the specific effects of different types of musical training on the EF/AP as well as on brain structure and function in the prefrontal area are still unclear.

## Neural Mechanisms for Active Musical Performance

Several neuroimaging/neurophysiological studies investigated the brain activity during active musical performance in musician. One study using near-infrared spectroscopy (NIRS) reported that piano playing engaged the frontal activity and playing more complex musical piece activated significantly wider frontal areas in musicians (Hashimoto and Okamoto, 2006). Furthermore, the brain activity during musical improvisation was investigated in amateur and professional musicians with varying experience of improvisation. These studies suggest that this creative musical activity extensively engages the brain activity in the prefrontal regions such as dorsal premotor area, pre-SMA, SMA, medial prefrontal cortex (mPFC), dorsolateral prefrontal cortex (DLPFC), anterior cingulate cortex (ACC), inferior frontal gyrus (IFG), and anterior insula (Brown et al., 2006; Pinho et al., 2014; Beaty, 2015; Erkkinen and Berkowitz, 2018; Loui, 2018). However, the directionality of improvisation-induced brain activity varied across the studies. The enhanced prefrontal brain activity and connectivity was interpreted as a reflection of goal-directed, top-down processing including motor planning, response selection, inhibition of competing stimuli, and conscious monitoring (Bengtsson, 2007; Villarreal et al., 2013). The dissociation of decreased lateral and increased medial prefrontal brain activity was interpreted as a reflection of a disconnection or disintegration of the lateral regions to suppress the top-down conscious control to generate spontaneous ideas, engaging the medial prefrontal regions, which was observed in more experienced improvisers (Limb and Braun, 2008; Liu et al., 2012). Further, the decreased lateral prefrontal activity and functional connectivity was interpreted as indicating that experts could spare EF load for the highly automatized activity (de Manzano and Ullén, 2012; Pinho et al., 2014; Rosen et al., 2016). The observed activation or deactivation in the lateral prefrontal cortex may also depend on the degree of top-down control required by a given improvisation task (Berkowitz and Ansari, 2008; Pinho et al., 2014). For example, the deactivation in the fronto-parietal regions was greater as the freedom of improvisation task increased (Berkowitz and Ansari, 2008). On the other hand, the enhanced lateral prefrontal activity observed in experienced improvisers may be due to the constrains imposed on the improvisation task (Bengtsson, 2007). In addition, the existing studies demonstrated that musical improvisation induced greater brain activity in the prefrontal regions such as DLPFC compared to simple musical repetitive or patterned tasks (Berkowitz and Ansari, 2008; Villarreal et al., 2013) and reproducing music (de Manzano and Ullén, 2012).

In summary, piano playing and musical improvisation extensively engages the prefrontal activity. This is likely because these musical activities involve EF/AP (Hannon and Trainor, 2007; Beaty, 2015). In addition, musical improvisation induces greater activation of the prefrontal regions and functional connectivity in musicians with less experience of improvisation and compared to simple musical repetitive and patterned tasks. Therefore, these musical activities may have some potential for EP/AP stimulation and neurorehabilitation.

## Music-Based Interventions for EF and AP Stimulation in Non-Musicians

There are a few studies that have demonstrated benefits of learning to play a musical instrument on EF in older healthy non-musicians. In one randomized control trial (RCT), 16 older adults received individualized piano lessons for 6 months (Bugos et al., 2007). They exhibited improvement on the Trail Making Test-B (TMT-B) over time during the period. This was not observed in a control group consisting of 15 older adults. In another study, a 4-month weekly group piano lessons designed and implemented by a professional music teacher and pianist resulted in improved performance on the Color-Word Stroop test in 13 older adults. This cognitive improvement was not observed in 16 older adults of the control group (Seinfeld et al., 2013). Another study further supported the benefit of group piano lessons on EF such as verbal flexibility and inhibition control in 24 older adults (Bugos, 2010). The EF improvement was significantly greater compared to a music appreciation group consisting of 22 older adults who had learned musical elements while listening to music. However, both music groups significantly improved the EF performance.

In addition, one EEG study with a pseudo-randomized design investigated the effects of music making on inhibition control and interference (Alain et al., 2019). Sixty healthy older non-musicians received either 3-month musical, visual art, or no training. The music-based intervention included music making using body percussion, and voice and non-pitched musical instruments, as well as learning basic music theory, and melody and harmony concepts by singing simple canons. It was provided by a professional music teacher. Transient differential neural activities were observed in the fronto-central sites in both intervention groups without showing improvement on the task performance.

In summary, learning to play the piano may have beneficial effects on EF in healthy older non-musicians. This may be because it is a complex process, requiring processes for the coordination of multiple sensory modalities, motor control, monitoring, working memory, inhibition, and attentional shifting (Hannon and Trainor, 2007; Seinfeld et al., 2013). However, the sample sizes of these studies are small with additional methodological limitations in some of the studies such as no randomization, no blind assessors, no active control group, and unknown intervention compliance.

## Music-Based Interventions for EF and AP Neurorehabilitation in Clinical Populations

Several clinical studies incorporated musical improvisation and instrument playing as their intervention techniques. A few pilot studies investigated the effects of Neurologic Music Therapy (NMT) training for EF/AP. It is guided by a NMT-certified music therapist and is based on group improvisation projects with a special emphasis on attention switching that requires participants to musically respond to specific musical cues (Gardiner and Thaut, 2014; Thaut and Gardiner, 2014). In a pseudo-experimental design (due to institutional constraints of U.S. Veterans Administration), 31 persons with acquired brain dysfunction received one 30-min NMT training session (Thaut et al., 2009). A paired *t*-test analysis showed that the music group significantly improved cognitive flexibility assessed on TMT-B with a large effect size (*d* = 1.21), but not memory functions. A control group (*N* = 23) without any intervention did not show any change on the test. In addition, the music group significantly increased their confidence in the EF skill. Other NMT studies included children/adolescents with neurodevelopmental disorders such as Attention Deficit Hyperactivity Disorder (Abrahams and van Dooren, 2018) and Autism Spectrum Disorder (Pasiali et al., 2014). In both studies, the interventions consisted of a 45-min weekly training session over a period of 6 weeks. Both studies reported some improvement on AP. However, both studies included small sample sizes (2 participants in the NMT group in the former study and 9 participants with varying severity in the latter study). Due to the methodological limitations, these NMT preliminary findings need to replicate in RCTs with large sample sizes.

In addition, one RCT included 35 older adults with Mini Mental State Examination scores ≥18 and employed a 12 bi-week group music cognitive training (Biasutti and Mangiacotti, 2018). The training was delivered by a specialist with music and neuropsychology background and consisted of voice and instrumental improvisation, which was created based on the framework of different music-based interventions including NMT (Thaut, 2005). Repeated ANOVA showed that the music group (*N* = 18) showed significant improvement in mental flexibility assessed on verbal fluency and showed a trend toward significance in selective attention assessed on Attentional Matrices test compared to an active control group (gymnastic activities; *N* = 17).

In another RCT, 28 chronic stroke patients without extensive musical experience received either 30-h of music-supported therapy (MST) or conventional physical training over a 10-week period (Fujioka et al., 2018). The MST protocol included mapping functional movements on playing musical instruments, which was based on the NMT technique (Thaut, 2005), as well as music-making with a therapist. The music group showed significant improvement in cognitive flexibility measured by TMT-B in the mid-intervention whereas the active control group showed the improvement post-intervention.

In one RCT combined with functional near-infrared spectroscopy (fNIRS), 39 persons with mild cognitive impairment were assigned either to an instructor-guided 12-week movement music therapy (MMT) or a comparable active non-musical control therapy (Shimizu et al., 2018). MMT included light exercises synchronized to the background music and playing a percussion instrument accompanied with familiar songs. The MMT group showed enhanced mPFC activity and increased functional connectivity within the prefrontal area compared to the control group. ANOVA did not show a significant group effect on the Frontal Assessment Battery score. This is likely because both groups showed some improvement post-intervention in which the MMT group showed a significant improvement while the active control group showed a trend toward significance, shown using a paired *t*-test. A major limitation of this study was that the number of participants in each group was imbalanced (MMT: *N* = 30 vs. control: *N* = 9).

There are also RCTs that demonstrated the beneficial effects of other music-based interventions on EF/AP. For example, a 12-week singing (four different familiar songs) intervention guided by a professional choir instructor led to a significant improvement on inhibitory control in 31 persons with mild Alzheimer’s disease (AD; Pongan et al., 2017). This positive effect was also observed in the active control group (painting) of 28 mild AD participants. Another RCT employed Sound Training for Attention and Memory in Dementia (STAM-Dem; Ceccato et al., 2012). STAM-Dem is a 12-week music-based manualized protocol delivered by a music therapist in which the participants perform specific movements to instructed sound stimuli that requires selective attention. It consists of multiple phases with increasing difficulty. Test score changes on selective attention were significantly greater (improved test score) in the music group of 27 participants compared to the control group of 23 participants who received standard care. In addition, 1-h daily listening to participant-selected favorite music guided by a music therapist for 2 months significantly improved AP recovery in 17 persons with acute post-stroke compared to both active and passive control groups of 19 and 17 participants, respectively (Särkämö et al., 2008). Furthermore, the AP improvement was also associated with a significant increase in the prefrontal gray matter volume in those with left hemisphere stroke (Särkämö et al., 2014).

In summary, clinical studies showed some potential of active music-based interventions such as musical improvisation and instrument playing-based activities to enhance EF/AP. However, their specific effectiveness is still unclear as active control interventions are often effective as well. In addition, there is a paucity of research literature with various clinical conditions. With few neuroimaging studies, it is still to be determined whether the music-based interventions have engaged executive control areas or whether neuroplasticity had occurred in those areas. Further, some studies show critical limitations in the research design such as a small sample size, no randomization, no active control condition, no follow-up assessment, no analysis of a group × time interaction effect, and no trained therapist.

## Future Directions and Perspectives for EF/AP Stimulation and Neurorehabilitation

The current paper summarizes the effects of formal musical training, active musical performance, and music-based interventions on EF/AP and associated brain activity in musicians, non-musicians, and clinical populations to present the future directions and perspectives of music-based interventions for the EF/AP stimulation and neurorehabilitation. Active musical performance such as piano playing and musical improvisation engages the prefrontal activity and some intervention studies showed the potential of these musical activities for a basis of the EF/AP stimulation and neurorehabilitation techniques. One study showed that enhanced inhibition control was associated with the extent of involvement of the musical activity in the motor system via the cognitive control system in musicians (Slater et al., 2017), suggesting that instrument playing may be more effective to enhance inhibition control compared to singing, for example. However, neuroimaging studies are needed to uncover the neural mechanisms for piano playing and musical improvisation in non-musicians and to demonstrate the direct link between these musical activities and the EF/AP performance. It is also useful for such studies to contrast images between musical activities and the resting state activity as well as between the two musical activities to show what areas are involved in each musical activity and how similar or different in the brain activity induced by these musical activities. Further, more RCTs with a rigorous design are warranted to replicate the findings. In addition, it is important to address whether these interventions are feasible and effective for different age groups and clinical populations with different clinical characteristics (e.g., severity, acute vs. chronic).

Furthermore, it is crucial to design music-based interventions that can tap into targeted processing components of EF/AP. Some of the clinical studies specifically targeted an enhancement of cognitive flexibility and selective attention, utilizing acoustic elements of music as cues or target stimuli (Thaut et al., 2009; Ceccato et al., 2012). However, as music is tightly connected to the attention system (Särkämö et al., 2008), it can temporally enhance cognitive performance associated with some of the AP as well as learning and memory via increased arousal (Hommel et al., 1990; Thaut et al., 2005; Thompson et al., 2006; Särkämö et al., 2008). Therefore, future RCTs should include music listening as an active control condition to determine the specific efficacy of active music-based interventions above the music-induced arousal effect on EF/AP with follow-up assessments.

Lastly, neuroimaging techniques should be combined to determine whether music-based interventions engage the brain activity of the executive control regions and networks as well as whether neuroplasticity has occurred in the expected regions and networks following the interventions. These studies are also useful to determine whether the observed patterns of hyper- or hypo-activations are due to compensation, neural efficiency, or attempted compensation (Grady, 2012). Such research studies can help growth in this domain, shedding an increasing light on the neural mechanisms of how music-based intervention techniques can tap into higher cognition to facilitate maintenance, enhancement, as well as recovery of cognitive functions.

## Author Contributions

YK conceived the concept of the manuscript. YK wrote the first draft of the manuscript and MT wrote the sections of the draft. Both authors contributed to manuscript revision, and read and approved the submitted version of the manuscript.

## Conflict of Interest

The authors declare that the research was conducted in the absence of any commercial or financial relationships that could be construed as a potential conflict of interest.
